# Time-dependent risk factors associated with the decline of estimated GFR in CKD patients

**DOI:** 10.1007/s10157-015-1132-0

**Published:** 2015-06-23

**Authors:** Wen-xiu Chang, Shigeyuki Arai, Yoshifuru Tamura, Takanori Kumagai, Tatsuru Ota, Shigeru Shibata, Yoshihide Fujigaki, Zhong-yang Shen, Shunya Uchida

**Affiliations:** Department of Internal Medicine, Teikyo University School of Medicine, 2-11-1 Kaga, Itabashi, Tokyo 173-8605 Japan; Department of Nephrology, Tianjin First Central Hospital, No. 24 Fukang Road, Nankai District, Tianjin, China; Support for Community Medicine Endowed Chair, Teikyo University School of Medicine, 2-11-1 Kaga, Itabashi, Tokyo 173-8605 Japan; Department of Organ Transplantation, Tianjin First Central Hospital, No. 24 Fukang Road, Nankai District, Tianjin, China

**Keywords:** Time-dependent parameter, Time-averaged value, Estimated glomerular filtration rate, Multivariate linear regression analysis: multivariate logistic regression analysis, Rapid progression

## Abstract

**Background:**

Targeting the modifiable risk factors may help halt the progression of CKD, thus risk factor analysis is better performed using the parameters in the follow-up. This study aimed to examine the time-dependent risk factors for CKD progression using time-averaged values and to investigate the characteristics of rapid progression group.

**Methods:**

This is a retrospective cohort study enrolling 770 patients of CKD stage 3–4. Time-dependent parameters were calculated as time-averaged values by a trapezoidal rule. % decline of estimated GFR (eGFR) per year from entry was divided to three groups: <10 % (stable), 10–25 % (moderate progression), and ≥25 % (rapid progression). Multivariate regression analyses were employed for the baseline and the time-averaged datasets.

**Results:**

eGFR decline was 2.83 ± 4.04 mL/min/1.73 m^2^/year (8.8 ± 12.9 %) in male and 1.66 ± 3.23 mL/min/1.73 m^2^/year (5.4 ± 11.0 %) in female (*p* < 0.001). % decline of eGFR was associated with male, proteinuria, phosphorus, and systolic blood pressure as risk factors and with age, albumin, and hemoglobin as protective factors using either dataset. Baseline eGFR and diabetic nephropathy appeared in the baseline dataset, while uric acid appeared in the time-averaged dataset. The rapid progression group was associated with proteinuria, phosphorus, albumin, and hemoglobin in the follow-up.

**Conclusion:**

These results suggest that time-averaged values provide insightful clinical guide in targeting the risk factors. Rapid decline of eGFR is strongly associated with hyperphosphatemia, proteinuria, and anemia indicating that these risk factors should be intervened in the follow-up of CKD.

## Introduction

CKD has become a big burden on health and economy in world wide. The search for the progression factors of CKD may help solve the problem. A wide variety of studies have explored the prognostic value of certain clinical and biochemical parameters for the renal outcomes of CKD patients [1–3]. Based on the evidence, clinical guidelines such as KDIGO recommend optimal blood pressure control and trying to reduce persistent proteinuria in addition to the supportive therapy for the original kidney diseases [4]. Other risk factors regarding the renal outcomes are numerous but controversial in the literature, including age, sex, obesity, hemoglobin, albumin, uric acid, potassium disorders, phosphorus disorders, metabolic acidosis, dyslipidemia, hematuria, inflammation, inappropriate lifestyles, geography, and various genetic factors.

In addition, the reason for the inconsistent results may be attributed to use of baseline values in the cohort studies. Although race, sex, age, body height, and original kidney disease cannot be modifiable, blood parameters such as hemoglobin, potassium, phosphorus, and uric acid may vary profoundly by advancement of CKD stage and by optimal treatments in the follow-up period. Risk factors yet to be established are not properly intervened probably because the target range of the risk factors in the follow-up lacks evidence. Taking these unsolved problems into consideration, in the present study, we have decided to use time-averaged values for time-dependent covariates. Moreover, we have attempted to explore the risk factors of most rapid progression of CKD since the trajectory of the clinical course of CKD patients attracts increasing attention from a viewpoint of mortality and morbidity [5].

## Materials and methods

### Retrospective historical cohort

This retrospective cohort study was approved by the institutional review board (IRB) in the Teikyo University Review Board #14-115 and was executed in accordance with the principle of the Helsinki Declaration. Written informed consent was waived after approval of IRB and the patient records and information were anonymized and de-identified prior to analysis.

**Fig. 1:**
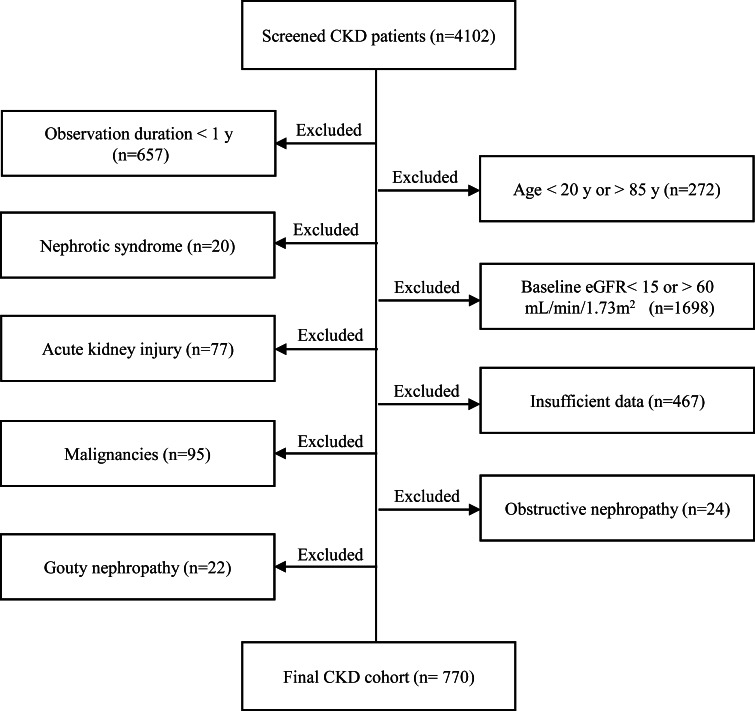
Flow chart of constructing retrospective CKD cohort

The CKD patients who visited between January 2008 and July 2013 in the Department of Nephrology, Teikyo University Hospital (Tokyo, Japan) were screened (*n* = 4102). The CKD patients ranging between stage 3 and 4 were followed until entering dialysis as a primary end point. Patients of age 20–84 years were included but patients having short observation period (less than 1 year), nephrotic syndrome, acute kidney injury, malignancies, obstructive nephropathy, and gouty nephropathy were excluded as shown in Fig. 1. Finally, 770 cases consisting of 481 males (62.5 %) and 289 females (37.5 %) were enrolled in this study.

### Parameters analyzed

The patients had regular follow-up visits at intervals of 1–3 months. The demographic characteristics included sex, age, body mass index, and original kidney disease (diabetic nephropathy or not). The information about antihypertensive drugs was recorded whether or not using angiotensin converting enzyme inhibitor or angiotensin II receptor blocker (both combined as RAS inhibitor; RASi), calcium channel blocker (CCB), diuretics or others.

Blood parameters were hemoglobin (Hb), albumin (Alb), urea nitrogen (UN), creatinine (Cr), uric acid (UA), Na, K, Cl, albumin-corrected Ca (cCa), inorganic phosphorus (P), low-density lipoprotein cholesterol (LDL-C), and C-reactive protein (CRP). Serum Na minus Cl was calculated as a surrogate of serum bicarbonate [6, 7]. Blood was examined using hematology autoanalyzer (Sysmex XE-5000, Kobe, Japan). The blood chemistry was measured by a routine measurement using autoanalyzer (LABOSPECT 008, Hitachi High-Technologies Corporation, Tokyo, Japan).

Urinary protein concentration measured by a pyrocatechol violet-metal complex assay method was expressed as gram per gram creatinine excretion (g/g Cr). The degrees of hematuria in dipstick were coded as four grades of 0–3 according to 0, 1+, 2+, and 3+, and as 0.5 if ±. Creatinine concentrations in serum and urine were measured by an enzymatic method after calibration based on isotope dilution mass spectrometry.

The eGFR was calculated using the Modification of Diet in Renal Disease (MDRD) study equation for Japanese population [8]; eGFR (mL/min/1.73 m^2^) = 194 × Cr^−1.094^ × Age^−0.287^ (if female, ×0.739). And the grade of CKD was classified based on the Kidney Disease Outcomes Quality Initiative (KDOQI) practice guidelines [9].

All the time-dependent parameters were calculated as time-averaged values based on a trapezoidal rule [1, 10]. In short, the area under curve formed by the repeated measurements at every visit was divided by the elapsed time up to the end of the follow-up period, yielding a single time-averaged value for a time-dependent parameter. When entered to dialysis, the calculation was stopped up to eGFR 5 mL/min/1.73 m^2^ to avoid the unstable state. Most of the patients were treated according to the guideline for CKD [11] and guidebook of CKD practice [12] originally based on KDOQI guideline.

### Renal outcome

The slope of the regression line divided by the baseline eGFR yielded % change of eGFR per year as a surrogate marker which was then divided to three groups; less than 10 % decline (as “stable” or Group 1), 10–25 % decline (as “moderate progression” or Group 2), and greater than or equal to 25 % decline of eGFR (as “rapid progression” or Group 3). These cut-off values were decided according to the precedent investigations [2, 13]. In the present study, initiation of dialysis therapy (either hemodialysis or peritoneal dialysis) was selected as a renal outcome.

### Statistical analyses

All continuous data are presented as mean ± standard deviation (SD). Comparison of continuous variables among three groups was analyzed by ANOVA followed by Tukey post hoc test. The Chi squared test was employed for the categorical variables. The difference between the baseline and time-averaged values was compared by paired *t* test, Wilcoxon signed-rank test or McNemar test as appropriate. Linear regression was performed by Pearson analysis after carefully checking multicollinearity. Multivariate linear regression analysis was carried out following a univariate linear regression analysis with *p* < 0.1 and the significant explanatory parameters were chosen in a stepwise forward manner. Multivariate logistic regression analysis was applied to detect the predictors of eGFR decline among three groups. The multivariate models used a stepwise forward selection procedure with *p* < 0.05 for entry and with *p* > 0.1 for removal of the covariate. The parameters for the rapid progression groups were subjected to receiver operating characteristic (ROC), showing the area under curve (AUC) with its 95 % confidence interval (95 % CI) and the cut-off point. All statistical analyses were performed using SPSS version 22 (IBM, Tokyo). A *p* < 0.05 was considered statistically significant.

## Results

### Demographic characteristics and baseline data

The demographic characteristics and baseline data are summarized in Table 1. The mean age was 61.9 ± 13.1 years old. The mean eGFR was 41.1 ± 13.2 mL/min/1.73 m^2^. Blood parameters fell within the respective normal ranges except for serum creatinine (1.44 ± 0.60 mg/dL). Spot urine samples revealed 0.99 ± 1.45 g/g Cr and the mean urine blood score was ± level corresponding to 0.52 ± 0.81.

**Table 1:** Clinical characteristics and laboratory data in the baseline dataset according to the decline of eGFR

Characteristics	Total (*n* = 770)	Group 1 (*n* = 561)	Group 2 (*n* = 137)	Group 3 (*n* = 72)	*p* value^¶^
Mean age (years)	61.9 ± 13.1	62.1 ± 13.0	62.1 ± 13.4	59.7 ± 14.0	0.330
Sex (M, %)	481 (62.5 %)	327 (58.3 %)	99 (72.3 %)**	55 (76.4 %)***	<0.001
eGFR_0 (mL/min/1.73 m^2^)	41.1 ± 13.2	44.5 ± 11.7	35.0 ± 12.3***	26.6 ± 12.0***^,†††^	<0.001
Original kidney disease					<0.001
DMN (%)	175 (22.7 %)	89 (15.9 %)	48 (35.0 %)	38 (52.8 %)	
HTN (%)	356 (46.2 %)	280 (49.9 %)	50 (36.5 %)	26 (36.1 %)	
CGN (%)	167 (21.7 %)	139 (24.8 %)	22 (16.1 %)	6 (8.3 %)	
Others (%)	72 (9.4 %)	53 (9.4 %)	17 (12.4 %)	2 (2.8 %)	
CKD stage					<0.001
3a (%)	352 (45.7 %)	310 (55.3 %)	32 (23.4 %)	10 (13.9 %)	
3b (%)	230 (29.9 %)	168 (30.0 %)	51 (37.2 %)	11 (15.3 %)	
4 (%)	188 (24.4 %)	83 (14.8 %)	54 (39.4 %)	51 (70.8 %)	
BMI_0 (kg/m^2^)	24.3 ± 4.4	24.2 ± 4.4	24.8 ± 4.4	24.6 ± 4.7	0.244
SBP_0 (mmHg)	137.5 ± 21.2	134.9 ± 20.0	141.9 ± 21.2	148.9 ± 24.8***^,††^	<0.001
Blood parameters					
Hb_0 (g/dL)	12.9 ± 1.9	13.2 ± 1.8	12.3 ± 1.9**	11.5 ± 2.0***^,†††^	<0.001
TP_0 (g/dL)	6.9 ± 0.6	7.0 ± 0.6	6.8 ± 0.6**	6.5 ± 0.6***^,†††^	<0.001
Alb_0 (g/dL)	4.0 ± 0.5	4.1 ± 0.4	3.8 ± 0.5**	3.5 ± 0.5***^,†††^	<0.001
BUN_0 (mg/dL)	23.0 ± 9.8	20.5 ± 7.5	27.3 ± 9.4***	34.5 ± 14.4***^,†††^	<0.001
Cr_0 (mg/dL)	1.44 ± 0.60	1.27 ± 0.44	1.70 ± 0.62***	2.28 ± 0.74***^,†††^	<0.001
UA_0 (mg/dL)	6.5 ± 1.4	6.3 ± 1.4	6.8 ± 1.3*	7.3 ± 1.6***^,†††^	<0.001
Na_0 (mEq/L)	140.7 ± 2.7	140.9 ± 2.7	140.3 ± 2.9	140.2 ± 2.2	0.029
K_0 (mEq/L)	4.45 ± 0.52	4.40 ± 0.47	4.63 ± 0.55	4.75 ± 0.61***^,†††^	<0.001
Cl_0 (mEq/L)	105.3 ± 3.2	105.0 ± 3.0	105.6 ± 3.5**	107.1 ± 3.4***	<0.001
Na-Cl_0 (mEq/L)	35.2 ± 3.6	35.6 ± 3.8	34.8 ± 2.5**	33.1 ± 3.1***^,†^	<0.001
cCa_0 (mg/dL)	8.9 ± 0.5	8.8 ± 0.5	8.9 ± 0.5	8.9 ± 0.5	0.151
P_0 (mg/dL)	3.4 ± 0.5	3.3 ± 0.5	3.5 ± 0.5**	3.7 ± 0.5***^,†††^	<0.001
CRP_0 (mg/dL)	0.2 ± 0.5	0.2 ± 0.5	0.3 ± 0.8*	0.2 ± 0.4	0.043
LDL-C_0 (mg/dL)	111.4 ± 30.4	113.4 ± 29.5	105.8 ± 30.6	106.0 ± 35.1^†^	0.009
Urine parameters (spot)					
TPU/CrU_0 (g/g Cr)	0.99 ± 1.45	0.56 ± 0.82	1.68 ± 1.75**	2.94 ± 2.32***^,†††^	<0.001
UB_score_0	0.52 ± 0.81	0.48 ± 0.81	0.62 ± 0.81*	0.60 ± 0.72^††^	<0.001
Antihypertensive drugs					
RASi_0 (%)	424 (55.1 %)	319 (56.9 %)	67 (48.9 %)	38 (52.8 %)	0.225
CCB_0 (%)	292 (37.9 %)	211 (37.6 %)	47 (34.3 %)	34 (47.2 %)	0.180
Diuretic_0 (%)	122 (15.8 %)	75 (13.4 %)	27 (19.7 %)	20 (27.8 %)**	0.003
Other AHD_0 (%)	54 (7.0 %)	34 (6.1 %)	11 (8.0 %)	9 (12.5 %)	0.115

Groups 1, 2, and 3 correspond to % decline of eGFR per year <10 %, 10–25 %, and >25 %, respectively

“0” following the parameter denotes the baseline value

*eGFR_0* estimated glomerular filtration rate, *DMN* diabetic nephropathy, *HTN* hypertensive nephropathy, *CGN* chronic glomerulonephritis, *SBP* systolic blood pressure, *Hb* hemoglobin, *TP* total protein, *Alb* albumin, *BUN* blood urea nitrogen, *Cr* creatinine, *UA* uric acid, *Na* sodium, *K* potassium, *Cl* chloride, *cCa* albumin-corrected calcium, *P* phosphorus, *CRP* C-reactive protein, *LDL-C* low-density lipoprotein cholesterol, *TPU/CrU* urine protein divided by urine creatinine, *UB_score* urine blood score, *RASi* RAS inhibitor, *CCB* calcium channel blocker, *AHD* antihypertensive drugs

^¶^ANOVA, Kruskal–Wallis H test (CRP, TPU/CrU, and UB_score) or cross table analysis (Sex, Original kidney disease, CKD stage, RASi, CCB, Diuretic, and other AHD) as appropriate

* *p* < 0.05, ** *p* < 0.01, *** *p* < 0.001 vs. Group 1; ^†^ *p* < 0.05, ^††^ *p* < 0.01, ^†††^ *p* < 0.001 vs. Group 2 (Tukey post-test or Mann–Whitney *U* test or cross table analysis)

Taken together, the baseline values stratified into the three groups showed some tendencies of the demographic characteristics; in Group 3, younger, male in preponderance, greater prevalence of diabetic nephropathy, higher grade of CKD stages, and higher SBP are shown (Table 1). Blood parameters also revealed lower hemoglobin, lower albumin, higher uric acid, higher potassium, higher chloride, lower Na-Cl, and higher phosphorus (Table 1). Urinary protein excretion and hematuria were greater in Groups 2 and 3 than Group 1. The mostly administered drug was RASi that was given to more than half of the patients (Table 1).

### Time-averaged values in the follow-up

**Fig. 2:**
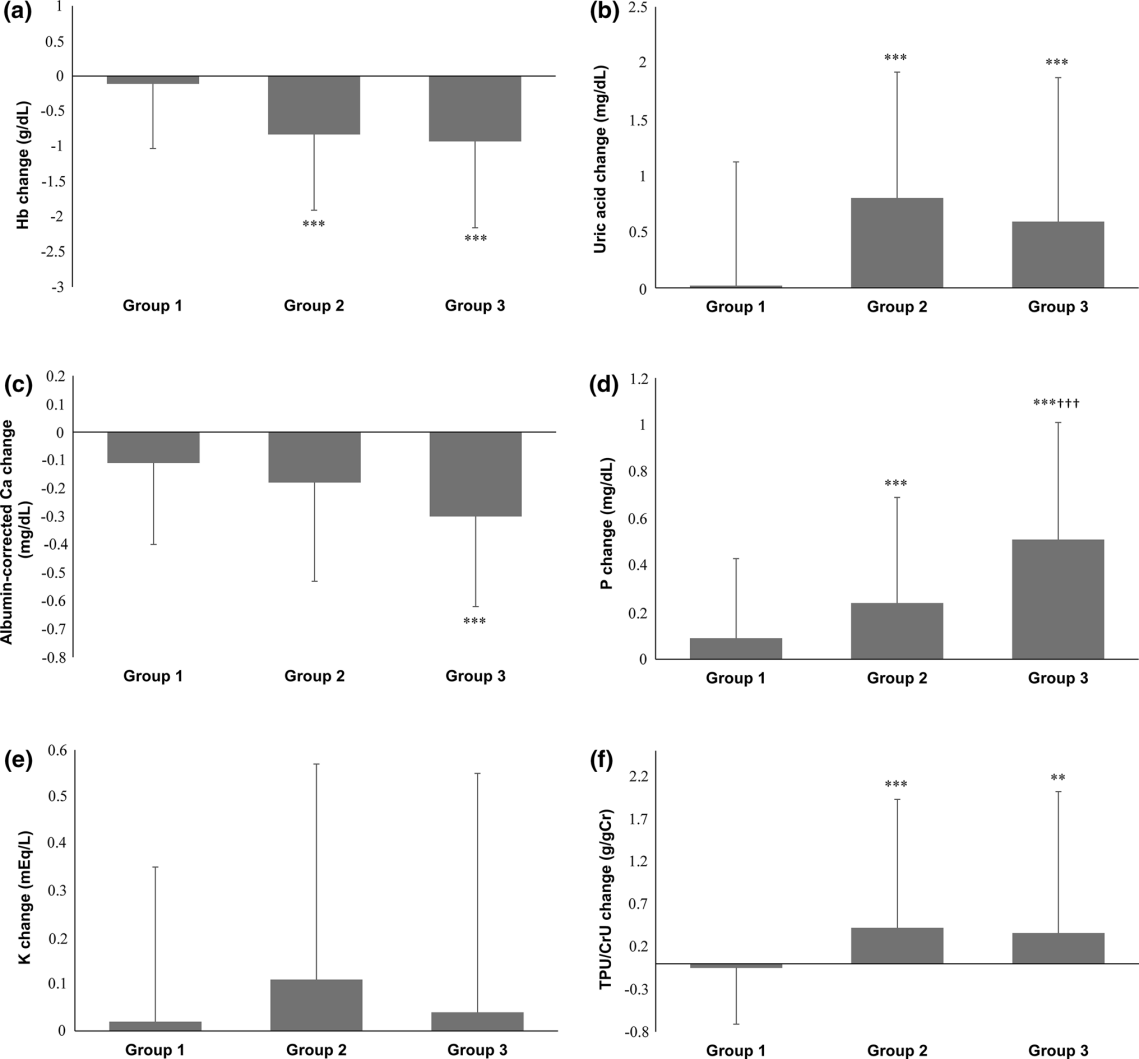
Changes from baseline values to time-averaged values according to three groups. Groups 1, 2, and 3 correspond to % decline of eGFR per year <10 %, 10–25 %, and >25 %, respectively. Hemoglobin and albumin-corrected Ca significantly decreased, whereas uric acid, phosphorus, potassium, and proteinuria significantly increased compared with baseline values. **p < 0.01, ***p < 0.001 vs. Group 1; ^†††^ *p* < 0.001 vs. Group 2 (ANOVA followed by Tukey post-test)

Follow-up data are shown in Table 2. The mean observation period was 4.1 ± 1.6 years. The mean % decline of eGFR per year was 7.5 ± 12.4 %, and by separating to three groups, 1.6 ± 4.7 % in Group 1, 15.8 ± 4.3 % in Group 2, and 37.7 ± 11.2 % in Group 3 (ANOVA, *p* < 0.001). 110 patients (14.3 %) out of 770 participants reached ESRD including hemodialysis (*n* = 89) and peritoneal dialysis (*n* = 21). Time-averaged values of the time-dependent parameters were compared with the baseline values in the total patients by paired *t* test or Wilcoxon signed-rank test as appropriate (Table 2). Systolic blood pressure, hemoglobin, Na-Cl, albumin-corrected Ca, LDL-C, and hematuria significantly decreased whereas uric acid, phosphorus, and proteinuria significantly increased in the time-averaged values (Table 2). The time-averaged characteristics of the three groups divided by % decline of eGFR per year are also summarized in Table 2. The changes between the baseline and time-averaged values are plotted and the statistical significances are shown against Group 1 or Group 2 (Fig. 2). As compared with Group 1, the significant changes were noticed in hemoglobin, uric acid, corrected Ca, phosphorus, potassium, CRP, and proteinuria in Groups 2 and 3. The result suggests that the changes of these parameters in Groups 2 and 3 are augmented in the follow-up and the dependency to the % decline of eGFR is pronounced in hemoglobin and phosphorus (Fig. 2).

**Table 2:** Time-averaged values in the time-averaged dataset according to the decline of eGFR

Characteristics	*p* value^§^ (vs. baseline)	Total (*n* = 770)	Group 1 (*n* = 561)	Group 2 (*n* = 137)	Group 3 (*n* = 72)	*p* value^¶^
Observation period (years)		4.1 ± 1.6	4.4 ± 1.5	4.0 ± 1.4***	2.0 ± 0.7***^,†^	<0.001
% decline of eGFR/year		7.5 ± 12.4	1.6 ± 4.7	15.8 ± 4.3***	37.7 ± 11.2***^,†††^	<0.001
Enter dialysis (%)		110 (14.3 %)	3 (0.5 %)	52 (38.0 %)***	55 (76.4 %)***^,†††^	<0.001
SBP_t (mmHg)	<0.001	133.1 ± 13.9	130.5 ± 12.6	138.1 ± 14.2^**^	144.3 ± 14.9***^,†††^	<0.001
Blood parameters						
Hb_t (g/dL)	<0.001	12.6 ± 1.8	13.1 ± 1.6	11.5 ± 1.6***	10.6 ± 1.4***^,†††^	<0.001
TP_t (g/dL)	<0.001	7.0 ± 0.5	7.1 ± 0.4	6.9 ± 0.5***	6.5 ± 0.6***^,†††^	<0.001
Alb_t (g/dL)	<0.001	4.0 ± 0.4	4.1 ± 0.3	3.8 ± 0.4***	3.5 ± 0.5***^,†††^	<0.001
BUN_t (mg/dL)	<0.001	26.7 ± 13.9	21.3 ± 8.7	37.6 ± 13.3***	48.5 ± 15.1***^,†††^	<0.001
Cr_t (mg/dL)	<0.001	1.88 ± 1.21	1.38 ± 0.61	2.79 ± 1.19***	4.08 ± 1.29***^,†††^	<0.001
UA_t (mg/dL)	<0.001	6.7 ± 1.3	6.3 ± 1.2	7.6 ± 1.0	7.9 ± 1.1***^,†††^	<0.001
Na_t (mEq/L)	0.875	140.7 ± 2.2	140.9 ± 2.2	140.3 ± 1.9	139.8 ± 2.5***^,†^	<0.001
K_t (mEq/L)	0.009	4.51 ± 0.44	4.42 ± 0.40	4.74 ± 0.47	4.79 ± 0.48***^,†††^	<0.001
Cl_t (mEq/L)	<0.001	105.7 ± 2.6	105.4 ± 2.4	106.2 ± 2.8	106.9 ± 3.3***^,††^	<0.001
Na-Cl_t (mEq/L)	<0.001	34.9 ± 3.4	35.3 ± 3.6	34.1 ± 2.4***	32.9 ± 2.0***^,†††^	<0.001
cCa_t (mg/dL)	<0.001	8.7 ± 0.4	8.7 ± 0.4	8.7 ± 0.4	8.6 ± 0.4	0.227
P_t (mg/dL)	<0.001	3.5 ± 0.5	3.4 ± 0.4	3.7 ± 0.5***	4.2 ± 0.6***^,†††^	<0.001
CRP_t (mg/dL)	<0.001	0.3 ± 0.4	0.2 ± 0.3	0.3 ± 0.4***	0.4 ± 0.4**	<0.001
LDL-C_t (mg/dL)	<0.001	104.6 ± 23.3	106.6 ± 22.7	99.8 ± 23.1	98.6 ± 26.0*^,††^	0.001
Urine parameters (spot)						
TPU/CrU_t (g/g Cr)	0.192	1.06 ± 1.43	0.51 ± 0.74	2.10 ± 1.50***	3.30 ± 1.94***^,††^	<0.001
UB_score_t	<0.001	0.40 ± 0.55	0.35 ± 0.56	0.48 ± 0.51	0.59 ± 0.51**	<0.001
Antihypertensive drugs						
RASi_t (%)	<0.001	626 (81.3 %)	449 (80.0 %)	114 (83.2 %)	63 (87.5 %)	0.254
CCB_t (%)	<0.001	491 (63.8 %)	326 (58.1 %)	107 (78.1 %)***	58 (80.6 %)***	<0.001
Diuretic_t (%)	<0.001	314 (40.8 %)	172 (30.7 %)	90 (65.7 %)***	52 (72.2 %)***	<0.001
Other AHD_t (%)	<0.001	184 (23.9 %)	107 (19.1 %)	50 (36.5 %)***	27 (37.5 %)**	<0.001

Groups 1, 2, and 3 correspond to % decline of eGFR per year <10 %, 10–25 %, and >25 %, respectively

“*t*” following the parameter denotes the time-averaged value or follow-up value

*eGFR* estimated glomerular filtration rate, *SBP* systolic blood pressure, *Hb* hemoglobin, *TP* total protein, *Alb* albumin, *BUN* blood urea nitrogen, *Cr* creatinine, *UA* uric acid, *Na* sodium, *K* potassium, *Cl* chloride, *cCa* albumin-corrected calcium, *P* phosphorus, *CRP* C-reactive protein, *LDL-C* low-density lipoprotein cholesterol, *TPU/CrU* urine protein divided by urine creatinine, *UB_score* urine blood score, *RASi* RAS inhibitor, *CCB* calcium channel blocker, *AHD* antihypertensive drugs

^§^ *p* values vs. baseline values in the total patients by paired *t* test, Wilcoxon signed-rank test (CRP, TPU/CrU, and UB_score) or McNemar (RASi, CCB, Diuretic, and other AHD)

^¶^ANOVA, Kruskal–Wallis *H* test (CRP, TPU/CrU, and UB_score) or cross table analysis (Enter dialysis, RASi, CCB, Diuretic, and other AHD)

* *p* < 0.05, ** *p* < 0.01, *** *p* < 0.001 vs. Group 1; ^†^ *p* < 0.05, ^††^ *p* < 0.01, ^†††^ *p* < 0.001 vs. Group 2 (ANOVA followed by Tukey post-test or cross table analysis)

*** *p* < 0.001 vs. Group 1; ^†††^ *p* < 0.001 vs. Group 2 (Mann–Whitney *U* test)

### Linear regression analysis with % change in eGFR per year in total patients

We examined a linear regression analysis using % decline of eGFR per year as a dependent parameter. Since baseline values and time-averaged values of the covariates showed strong linearity, the statistical analysis was carried out separately using the baseline dataset or the time-averaged dataset to avoid multicollinearity.

First, a univariate linear regression between baseline values and % change in eGFR was performed. 19 parameters including age, sex, eGFR_0, DMN, BMI_0, SBP_0, Hb_0, Alb_0, UA_0, Na_0, K_0, Na-Cl_0, cCa_0, P_0, CRP_0, LDL-C_0, TPU/CrU_0, UB_score_0, RASi_0 were examined one by one as an explanatory covariate using a univariate linear regression analysis with exclusion cut-off of *p* > 0.1. Then a multivariate linear regression analysis extracted significant independent parameters as shown in Table 3. In a descending order of the significance, proteinuria, baseline eGFR, albumin, phosphorus, male, age, systolic blood pressure, DMN, and hemoglobin were selected in total patients (Table 3). Adjusted coefficient of determination turned out to be 42 %.

**Table 3:** Linear regression with % change in eGFR per year in the baseline dataset

CKD stage	Characteristics	*β*	95 % CI	Adjusted *R* ^2^	*t* value	*p* value
Total (*n* = 770)	TPU/CrU_0	2.61	2.05 to 3.16	0.26	−9.24	<0.001
	eGFR_0	−0.21	−0.27 to −0.15	0.35	−6.87	<0.001
	Alb_0	−3.84	−5.60 to −2.08	0.37	−4.28	<0.001
	P_0	2.93	1.49 to 4.36	0.38	4.00	<0.001
	Sex (M)	3.43	1.89 to 4.96	0.40	4.37	<0.001
	Age	−0.11	−0.17 to −0.06	0.40	−4.17	<0.001
	SBP_0	0.04	0.01 to 0.08	0.41	2.58	0.010
	DMN	2.16	0.44 to 3.88	0.41	2.47	0.014
	Hb_0	−0.57	−1.03 to −0.10	0.42	−2.40	0.017
3a (*n* = 352)	TPU/CrU_0	2.37	1.64 to 3.10	0.20	6.34	<0.001
	DMN	2.84	1.01 to 4.67	0.25	3.05	0.002
	Sex (M)	4.78	3.26 to 6.30	0.28	6.19	<0.001
	Hb_0	−1.05	−1.57 to −0.54	0.32	−4.02	<0.001
	Age	−0.09	−0.14 to −0.04	0.34	−3.53	<0.001
	Alb_0	−2.31	−4.09 to −0.54	0.35	−2.56	0.011
	SBP_0	0.04	0.00 to 0.07	0.35	2.22	0.027
	P_0	1.55	0.08 to 3.01	0.36	2.08	0.038
3b (*n* = 230)	TPU/CrU_0	2.53	1.82 to 3.23	0.25	7.03	<0.001
	DMN	4.99	2.43 to 7.55	0.30	3.84	<0.001
	Alb_0	−3.55	−6.07 to −1.04	0.31	−2.78	0.006
	Age	−0.09	−0.17 to −0.01	0.32	−2.21	0.028
4 (*n* = 188)	TPU/CrU_0	3.35	2.11 to 4.59	0.22	5.33	<0.001
	eGFR_0	−1.29	−1.76 to −0.82	0.32	−5.44	<0.001
	Alb_0	−8.21	−13.11 to −3.31	0.35	−3.30	<0.001
	Age	−0.19	−0.36 to −0.02	0.36	−2.20	0.029

Independent variables included age, sex, eGFR_0, DMN, BMI_0, SBP_0, Hb_0, Alb_0, UA_0, Na_0, K_0, Na-Cl_0, cCa_0, P_0, CRP_0, LDL-C_0, TPU/CrU_0, UB_score_0, RASi_0

Next, the time-averaged dataset was analyzed in the same way with % change in eGFR as a dependent covariate. A multivariate linear regression analysis disclosed proteinuria, phosphorus, uric acid, hemoglobin, male, systolic blood pressure, age, and albumin in a descending order of the significance in total participants (Table 4). Adjusted coefficient of determination was 60 % which increased from 42 %.

**Table 4:** Linear regression with % change in eGFR per year using the time-averaged dataset

CKD stage	Characteristics	*β*	95 % CI	Adjusted *R* ^2^	*t* value	*p* value
Total (*n* = 770)	TPU/CrU_t	2.77	2.22 to 3.31	0.41	10.00	<0.001
	P_t	5.35	4.02 to 6.67	0.49	7.91	<0.001
	UA_t	1.68	1.19 to 2.17	0.55	6.76	<0.001
	Hb_t	−1.49	−1.91 to −1.07	0.57	−7.03	<0.001
	Sex (M)	3.39	2.08 to 4.71	0.59	5.07	<0.001
	SBP_t	0.09	0.04 to 0.13	0.59	3.89	<0.001
	Age	−0.07	−0.11 to −0.02	0.59	−2.80	0.005
	Alb_t	−2.52	−4.38 to −0.65	0.60	−2.65	0.008
3a (*n* = 352)	TPU/CrU_t	1.78	1.18 to 2.38	0.34	5.85	<0.001
	UA_t	1.37	0.83 to 1.90	0.42	5.02	<0.001
	Hb_t	−1.49	−1.92 to −1.05	0.49	−6.71	<0.001
	Sex (M)	3.05	1.69 to 4.40	0.52	4.43	<0.001
	Na_t	−0.35	−0.64 to −0.06	0.53	−2.36	0.019
	Alb_t	−3.68	−5.61 to −1.75	0.54	−3.74	<0.001
	SBP_t	0.09	0.04 to 0.13	0.55	3.74	<0.001
	Age	−0.07	−0.11 to −0.02	0.56	−2.83	0.005
3b (*n* = 230)	TPU/CrU_t	3.32	2.68 to 3.96	0.44	10.21	<0.001
	UA_t	2.01	1.30 to 2.73	0.50	5.54	<0.001
	Hb_t	−1.11	−1.63 to −0.59	0.54	−4.18	<0.001
	P_t	2.99	0.97 to 5.00	0.56	2.93	0.004
4 (*n* = 188)	TPU/CrU_t	4.53	3.32 to 5.74	0.37	7.40	<0.001
	P_t	7.50	4.08 to 10.92	0.48	4.32	<0.001
	UA_t	2.53	1.19 to 3.86	0.52	3.74	<0.001
	Hb_t	−1.90	−3.13 to −0.67	0.54	−3.05	0.003
	Sex (M)	3.88	0.17 to 7.60	0.55	2.06	0.041

Independent variables included age, sex, eGFR_0, DMN, SBP_t, Hb_t, Alb_t, UA_t, Na_t, K_t, Na-Cl_t, cCa_t, P_t, CRP_t, LDL-C_t, TPU/CrU_t, UB_score_t, RASi_t

It is appreciated that proteinuria had been always the most powerful impact on eGFR decline. Subsequently, male, phosphorus, systolic blood pressure followed both at entry and in the follow-up whereas age, hemoglobin, and albumin had protective effects on CKD progression.

### Linear regression analysis with % change in eGFR per year depending on CKD stages

The analysis was reiterated according to CKD stages. It is striking that proteinuria was invariably included in any stage of CKD as a strong risk factor. Male, hemoglobin, systolic blood pressure, and phosphorus disappeared in CKD stage 3b or 4 (Table 3). The patients with CKD 4 were affected by proteinuria, baseline eGFR, albumin, and age in the kidney progression (Table 3). Similarly, influential factors in the follow-up constituted proteinuria, uric acid, hemoglobin, and phosphorus in the CKD stage 3b and proteinuria, phosphorus, uric acid, hemoglobin, and male in CKD stage 4 (Table 4). Of note is that uric acid was always included in the results using time-averaged dataset according to CKD stages. In other words, without analyzing the time-averaged uric acid, it is likely to overlook such a result.

**Fig. 3:**
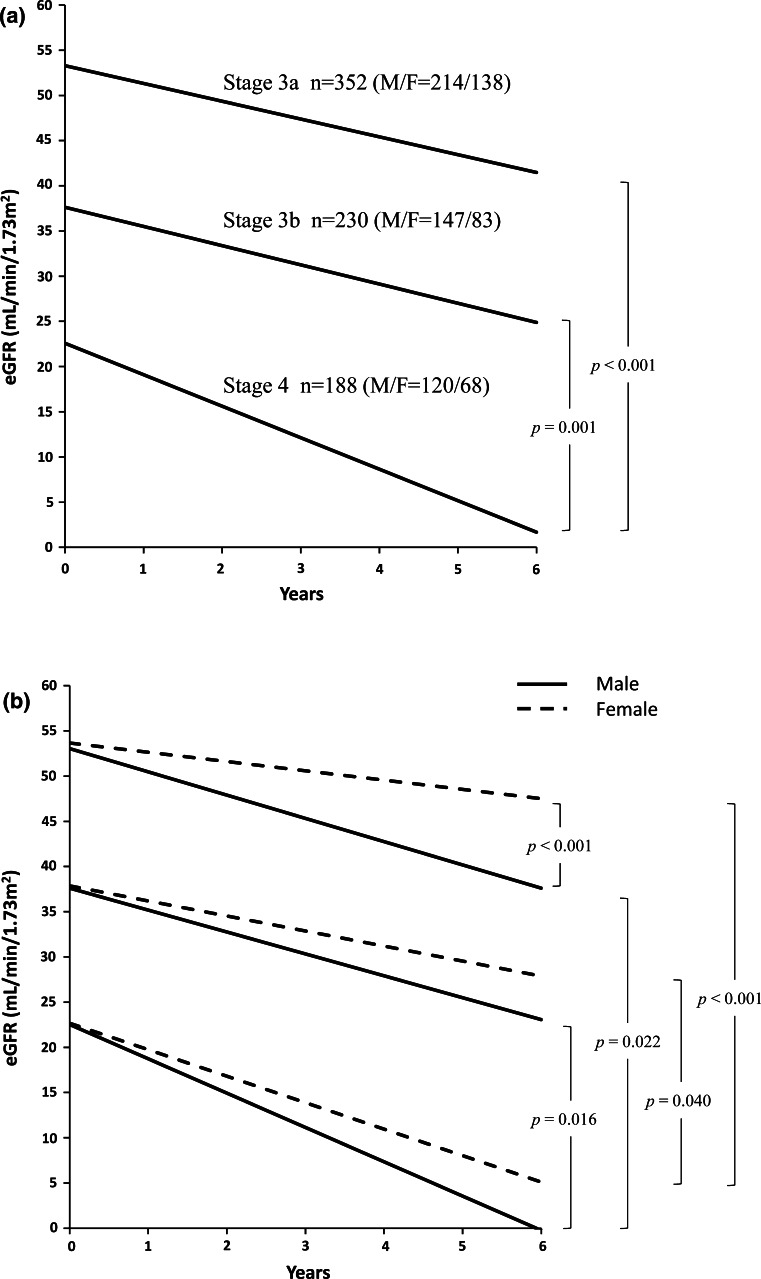
The slope of eGFR decline according to CKD stages. **a** The slope of eGFR decline in total patients. *M* male, *F* female. *p* < 0.001 (ANOVA followed by Tukey post-test). **b** Sex difference in the slope of eGFR decline. The slope of stage 4 was significantly steeper than that of stage 3a or 3b (ANOVA followed by Tukey post-test). The difference between male and female was seen in stage 3a, *p* < 0.001 (unpaired *t* test)

The slopes of the eGFR decline per year were compared according to CKD stages in the total patients and in the patients separated by sex (Table 5). The slopes between CKD stage 3a and 3b did not show the statistical significance in the total patients, nor so in the subgroups separated by sex. However, the slope of the patients with CKD stage 4 demonstrated significantly steeper lines in the total patients (Fig. 3a). When separated by sex, the eGFR decline in male patients showed steeper than that of female in any stage of CKD, though the statistical significance was only attained in CKD stage 3a (Fig. 3b).

**Table 5:** eGFR decline per year according to CKD stage

CKD stage	Total		Male		Female	
	*n*	eGFR decline/year (mL/min/1.73 m^2^/year)	*n*	eGFR decline/year (mL/min/1.73 m^2^/year)	*n*	eGFR decline/years (mL/min/1.73 m^2^/years)
3a	352	1.97 ± 4.00	214	2.58 ± 4.53	138	1.03 ± 2.74^‡^
3b	230	2.15 ± 3.49	147	2.42 ± 3.60	83	1.66 ± 3.24
4	188	3.48 ± 3.60***^,††^	120	3.79 ± 3.48*^,†^	68	2.93 ± 3.77***^,†^

* *p* < 0.05, *** *p* < 0.001 vs. CKD stage 3a; ^†^ *p* < 0.05, ^††^ *p* < 0.01 vs. CKD stage 3b (ANOVA, Tukey post hoc test)

^‡^ *p* < 0.001 vs. male by unpaired *t* test

### Logistic regression in total patients

A multivariate logistic regression analysis showed that the rapid progression group (Group 3) was associated with baseline parameters such as eGFR, proteinuria, albumin, and phosphorus in addition to age and male as compared with stable and moderate progression groups (Table 6). Using the time-averaged dataset, the rapid progression group was associated with proteinuria, phosphorus, albumin, and hemoglobin in the follow-up in addition to baseline eGFR and male (Table 7). The cut-off values for indicating the rapid progression of eGFR were proteinuria >1.39 g/g Cr, phosphorus >3.82 mg/dL, albumin <3.86 g/dL, and hemoglobin <11.6 g/dL in the follow-up (Table 8).

**Table 6:** Multivariate logistic regression among three groups according to % change in eGFR in the baseline dataset (*n* = 770)

Reference	Group	Characteristics	*β*	Wald	Exp(*β*)	95 % CI	*p* value
Group 1 (*n* = 561)	Group 2 (*n* = 137)	TPU/CrU_0	0.62	36.82	1.86	1.52 to 2.28	<0.001
		eGFR_0	−0.05	28.07	0.95	0.94 to 0.97	<0.001
		Alb_0	−1.00	15.13	0.37	0.22 to 0.61	<0.001
		Sex (M)	0.85	11.71	2.33	1.44 to 3.78	0.001
		P_0	0.77	11.06	2.16	1.37 to 3.39	0.001
	Group 3 (*n* = 72)	TPU/CrU_0	0.89	54.67	2.43	1.92 to 3.08	<0.001
		eGFR_0	−0.11	47.51	0.89	0.86 to 0.92	<0.001
		P_0	1.74	22.91	5.70	2.79 to 11.62	<0.001
		Alb_0	−1.64	19.97	0.19	0.09 to 0.40	<0.001
		Sex (M)	1.38	12.26	3.98	1.84 to 8.62	<0.001
Group 2	Group 3	eGFR_0	−0.07	17.42	0.94	0.91 to 0.97	<0.001
		TPU/CrU_0	0.27	10.69	1.31	1.11 to 1.53	0.001
		P_0	0.97	7.59	2.64	1.32 to 5.27	0.006
Group 1 + 2	Group 3	eGFR_0	−1.00	39.42	0.91	088 to 0.93	<0.001
		TPU/CrU_0	0.45	28.55	1.57	1.33 to 1.88	<0.001
		Alb_0	−1.31	15.03	0.27	0.14 to 0.52	<0.001
		P_0	1.23	12.79	3.44	1.75 to 6.76	<0.001
		Age	−0.31	6.29	0.97	0.95 to 0.99	0.012
		Sex (M)	0.85	5.23	2.35	1.13 to 4.88	0.022

Groups 1, 2, and 3 correspond to % decline of eGFR per year <10 %, 10–25 %, and >25 %, respectively

Independent variables included age, sex, eGFR_0, DMN, BMI_0, SBP_0, Hb_0, Alb_0, UA_0, Na_0, K_0, Na-Cl_0, cCa_0, P_0, CRP_0, LDL-C_0, TPU/CrU_0, UB_score_0, RASi_0

**Table 7:** Multivariate logistic regression among three groups according to % change in eGFR in the time-averaged dataset (*n* = 770)

Reference	Group	Characteristics	*β*	Wald	Exp(*β*)	95 % CI	*p* value
Group 1 (*n* = 561)	Group 2 (*n* = 137)	TPU/CrU_t	0.92	59.61	2.50	1.98 to 3.16	<0.001
		Hb_t	−0.53	34.97	0.59	0.49 to 0.70	<0.001
		UA_t	0.71	32.94	2.04	1.60 to 2.60	<0.001
		Sex(M)	1.04	10.47	2.82	1.51 to 5.28	0.001
		P_t	1.03	9.60	2.79	1.46 to 5.35	0.002
		CRP_t	0.74	5.51	2.09	1.13 to 3.85	0.019
	Group 3 (*n* = 72)	TPU/CrU_t	1.33	74.77	3.78	2.79 to 5.10	<0.001
		Hb_t	−0.93	35.05	0.40	0.29 to 0.54	<0.001
		P_t	2.80	32.95	16.41	6.31 to 42.64	<0.001
		UA_t	0.86	19.45	2.36	1.61 to 3.45	<0.001
		Sex(M)	1.90	14.94	6.71	2.56 to 17.61	<0.001
		CRP_t	1.25	6.51	3.48	1.34 to 9.08	0.011
Group 2	Group 3	P_t	1.77	19.75	5.88	2.69 to 12.83	<0.001
		TPU/CrU_t	0.41	14.77	1.51	1.22 to 1.86	<0.001
		Hb_t	−0.40	8.09	0.67	0.51 to 0.88	0.004
		Sex(M)	0.87	4.18	2.38	1.04 to 5.46	0.041
Group 1 + 2	Group 3	TPU/CrU_t	0.58	19.84	1.78	1.38 to 2.29	<0.001
		P_t	1.87	19.47	6.49	2.83 to 14.89	<0.001
		eGFR_0	−0.05	7.67	0.95	0.92 to 0.99	0.006
		Sex(M)	1.15	7.56	3.17	1.39 to 7.21	0.006
		Alb_t	−1.33	7.02	0.26	0.10 to 0.71	0.008
		Hb_t	−0.38	6.36	0.68	0.51 to 0.92	0.012

Groups 1, 2, and 3 correspond to % decline of eGFR per year <10 %, 10–25 % and >25 %, respectively

Independent variables included age, sex, eGFR_0, DMN, SBP_t, Hb_t, Alb_t, UA_t, Na_t, K_t, Na-Cl_t, cCa_t, P_t, CRP_t, LDL-C_t, TPU/CrU_t, UB_score_t, RASi_t

**Table 8:** Cut-off points for associating with rapid progression group (>25 % decline of eGFR per year) in the baseline and time-averaged parameters (*n* = 770)

Parameters	Characteristics	AUC	95 % CI	Cut-off point
Baseline values	TPU/CrU_0	0.85	0.80 to 0.90	1.30 g/g Cr
	eGFR	0.83	0.77 to 0.88	23.8 mL/min/1.73 m^2^
	P_0	0.72	0.66 to 0.78	3.63 mg/dL
Time-averaged values	TPU/CrU_t	0.89	0.86 to 0.93	1.39 g/g Cr
	P_t	0.87	0.83 to 0.91	3.82 mg/dL
	Alb_t	0.82	0.76 to 0.87	3.86 g/dL
	Hb_t	0.82	0.76 to 0.87	11.6 g/dL

*AUC* area under curve

## Discussion

The search for the predictors of CKD progression has attracted a great deal of attention for the last decade in conjunction with the CKD movement worldwide. Until now, a line of evidence suggests that proteinuria, hypertension, anemia, and preceding kidney dysfunction may be the most influential predictors for CKD progression [1–3]. These results were confirmed by a larger scale of the cohort of Insurance Company in the United States [14]. Following these major risk factors, diabetes mellitus, arteriosclerosis, congestive heart failure, and hyperuricemia were unveiled as the second line of risk factors for ESRD [14, 15].

The present study was able to show confirmatory results as follows. First, the degree of proteinuria ranked the first grade risk factors for dialysis initiation. Subsequent to proteinuria, time-averaged parameters such as phosphorus, uric acid, and hemoglobin were the second group of risk factors of CKD progression. Although the results of the analysis are the association study, the implication of higher phosphorus, higher uric acid, and lower hemoglobin can easily be understood as significant risk factors. The impact of anemia was vividly dictated in the present study.

The present retrospective study also unveiled possible risk factors that have been reported previously but did not reach high evidence level due to a lack of consistency. Among them uric acid, phosphorus, albumin, and C-reactive protein are just the several predictors that the present investigation was able to find out. Time-dependent covariates are subjected to change over time due to the natural course of CKD and appropriate treatments. Proteinuria, however, withstands the treatments as judged by the marginal change of the degree of proteinuria (Tables 1, 2). In this Discussion, we focus on the significance of other risk factors besides proteinuria, blood pressure, or hemoglobin because these three risk factors already obtain firm consensus [5].

First, uric acid was found significant risk factors only when assessed in time-averaged values. Many observation studies published positive relationship between serum uric acid and CKD progression originated from the study by Iseki et al. [16]. Since then, many observational cohort studies suggested the strong association between hyperuricemia and CKD progression [1, 17, 18]. More recently, a meta-analysis about the risk of hyperuricemia and the progression of CKD was published but the authors concluded that the evidence was still scarce and a definite conclusion cannot be drawn [19]. The inconsistency is in part due to the use of the baseline uric acid in the previous studies. In fact, the effect of uric acid was not evident in the baseline dataset in the present study but extracted as a significant risk factor when using the time-averaged dataset, suggesting that the increase in uric acid in the follow-up might influence on the renal outcome. An on-going randomized clinical trial will clarify the effect of uric acid-lowering agents on kidney function decline in the near future.

Of note serum phosphorus has been selected as a strong risk factor especially in the logistic regression. Most rapid progression group was associated with proteinuria, phosphorus, albumin, and anemia in the follow-up as well as baseline eGFR and male, and other factors were all negated. The finding encourages to targeting the time-dependent risk factors. Regardless of the merely intimate association, it is safe to say that strictly controlling serum phosphorus in the follow-up may inhibit the rapid progression of CKD. The mechanism of higher phosphorus may lie in arterial sclerosis due to the deposition of calcium phosphate in the vessel walls [20] and other sequences of events may comprise the osteopontin-induced tubulointerstitial fibrosis and the increase in serum fibroblast growth factor 23 [21].

The influence of age was that the younger the age, the more rapidly progress the kidney function. The result seems paradoxical but may be reasonable in view of the speculation that younger kidney may have a room for rapid decline and that the older kidney has overcome several challenges in a long life thus rather can tolerate against pressure. In fact, several reports exist that younger patients are prone to progress to ESRD rather than elderly [22, 23].

Male appeared as a strong predictor of rapid eGFR decline in linear and logistic regression analyses by both baseline and time-averaged datasets. The influence of male was remarked especially in the lower eGFR such as CKD stage 4. The slope of eGFR decline in any CKD stage tended to be steeper in male than female in the present study. This result is consistent with the previous meta-analysis using 68 studies [24]. They concluded that men with chronic renal disease of various etiologies show a more rapid decline in renal function with time than do women [24]. The mechanism of the sex difference in the CKD progression needs to be explored because sex difference may be confounded with other risk factors such as arteriosclerosis.

The advantages of using time-averaged values in a search for predictor analysis are of several folds. First of all, the continued pressure against the target organ by risk factors can be properly assessed in a long-term manner. In CKD clinic, multidisciplinary treatments are recommended because the treatment one by one can delay the progression of CKD [4]. The results by time-averaged values can provide an insightful clue for intervention. Second, it is easy to understand for clinician why and how to treat patients. Not only attending physicians but also patients can accept the aim and goal of the respective treatment. Third, since the measurement at every visit can be combined as a single value, it can solve the problem of missing data if any. However, the work is tremendously laborious and needs intense work. Finally, it meets the recent trend to investigate the longitudinal follow-up data rather than the baseline data because time-dependent parameters may widely change in the magnitude in the follow-up. The analysis using time-dependent covariates may open the new horizon of the CKD research. In the present study, we could unveil the many risk factors so far alleged but not confirmed. It was made possible with the aid of time-dependent dataset by accumulating the medical files that measured blood and urine samples at every visit.

Nonetheless, the present study has several limitations. First of all, the retrospective cohort in the present study was derived from a single institute. Thus, the results cannot be generalized because it may cause selection bias and treatment bias in the study. Second, the number of the patients is still small especially when dividing to several subgroups. Third, other covariates such as CKD-MBD parameters or genetic factors were not under assessment in this cohort. Finally, the history of drug use was not collected except for antihypertensive drugs. Albeit these limitations, the present study was able to create the robust database of the retrospective cohort and to validate the alleged risk factors, suggesting that many modifiable progression and protection factors should be strictly intervened. It is expected that the results may help treat CKD patients in the clinical practice.

## Conclusion

Proteinuria, blood pressure, and anemia are the three major risk factors of CKD not only in the baseline but also in the follow-up. Uric acid and phosphorus are emerging risk factors in the clinical course of CKD, indicating that the appropriate intervention may retard the progression of CKD. Time-dependent covariates should better be evaluated as time-averaged values, allowing the physicians to obtain insightful clinical guidance.
